# Looking into the shadow: the eugenics argument in debates on reproductive technologies and practices

**DOI:** 10.1007/s40592-018-0086-x

**Published:** 2018-12-07

**Authors:** Giulia Cavaliere

**Affiliations:** Department of Global Health and Social Medicine, School of Global Affairs, King’s College London, Room 3.12, Bush House (NE), 30, Aldwych, London, WC2B 4BG UK

**Keywords:** Eugenics, Reproductive technologies, Coercion, Stigmatisation, Disability

## Abstract

Eugenics is often referred to in debates on the ethics of reproductive technologies and practices, in relation to the creation of moral boundaries between acceptable and unacceptable technologies, and acceptable and unacceptable uses of these technologies. Historians have argued that twentieth century eugenics cannot be reduced to a uniform set of practices, and that no simple lessons can be drawn from this complex history. Some authors stress the similarities between past eugenics and present reproductive technologies and practices (what I define throughout the paper as ‘the continuity view’) in order to condemn the latter. Others focus on the differences between past and present practices (what I define throughout the paper as ‘the discontinuity view’) in order to defend contemporary reproductive technologies. In this paper, I explore the meanings of the word ‘eugenics’ and the relationship between its past and present uses in terms of contemporary debates on reproductive technologies and practices. I argue that moral disagreement about present technologies originate in divergent views of condemnable and justifiable features of the past.

## Introduction

New assisted reproductive technologies such as mitochondrial replacement techniques (MRTs), reproductive screening technologies such as pre-implantation genetic diagnosis (PGD), pre-natal diagnosis (PND) and non-invasive prenatal testing (NIPT), as well as gene editing technologies such as CRISPR (Clustered Regularly Interspaced Short Palindromic Repeats) incite ethical controversies.^1^ They do so because procreating and raising children, and influencing the type and number of people who will inhabit our planet in the future, touch upon people’s core moral beliefs and values. Partly for this reason, assisted reproductive technologies and practices engender moral disagreement and give rise to many highly controversial debates in bioethics. Examples of the questions discussed within these debates include whether or not technologies will bring about better or worse states of affairs compared to the status quo; whether their introduction will cause increased injustice, discrimination, sexism, ableism and racism; or whether they will make our lives (or our children’s lives) happier, healthier and/or longer. Some arguments focus on the consequences, and others concern the intrinsic goodness or wrongness of these technologies and their applications.

While the ethical questions discussed in these debates in academia, the media and other public fora are fairly diverse, one set of these questions has a common and recurrent feature: eugenics. This set of questions includes whether a given technology is eugenic, whether it might bring eugenics back, and whether this possibility is something to be feared or welcomed. What is referred to as the “shadow of eugenics” (Buchanan et al. 2001, p. 27)—namely the collective memory of condemned practices such as forced sterilisations as well as the condemned science of heredity, shared systems of belief, policies and ideas of different actors—continues to permeate today’s ethical debates on reproductive technologies and practices. As I show in this paper, some authors stress elements of discontinuity between past eugenics and contemporary reproductive technologies and practices, while others focus on elements of continuity between past and present. Both groups agree on the wrongness of past eugenics, but they have different views on the relationship between past and present, and especially on the ethical standing of present technologies and practices. Authors who hold what I refer to as the “discontinuity view” between past and present defend reproductive technologies and practices, grounding some of their arguments in the differences between the latter technologies and the eugenic past; while those holding what I refer to as the “continuity view” condemn these technologies and practices, their arguments grounded in similarities with the past.

The content of the arguments underlying the discontinuity view varies slightly, but their form can be summarised as follows:

“Eugenics was intrinsically wrong because it entailed x, y, z; other things being equal, reproductive technologies and practices are not wrong because they lack x, y, z”.

Similarly, the content of the arguments of scholars who hold the continuity view varies slightly, but their form is homogeneous:

“Eugenics was intrinsically wrong because it entailed x, y, z;, reproductive technologies and practices are likewise wrong because they similarly have elements of x, y, z”.

Considering that arguments drawing on the discontinuity and continuity between past and present are subsumed in the ethical assessments of reproductive technologies and practices, one would expect a knowledge of both the past and the present to play an important role in such assessments. In other words, considering that both arguments heavily rely on ‘x, y, z’, i.e. on problematic features of past eugenics, to ground their condemnations or absolutions of reproductive technologies, one would expect their assessments to be supported by sound and detailed historical analyses.^2^ However, this is not entirely the case. As I show in this paper, what authors consider the capital sins of past eugenics vary greatly, and many of their arguments about both the past and the present are not based on in-depth historical analyses (Bashford 2010; Paul 1998). Past eugenics is assumed to be something despicable that ought not to be repeated, but those who participate in debates on the ethics of reproductive technologies and practices often fail to explicitly refer to what was wrong with eugenics and why.^3^ Furthermore, these arguments rely on accounts of the history of eugenics often limited to the practices carried out during Nazism, and to racist and coercive dimensions of eugenics policies and practices (Bashford 2010). Why is this the case? One potential answer is that there is a division of “*cognitive* labour” among academics ([emphasis in original] Kitcher 2011, p. 193), and: “a group of investigators, addressing a common problem, pursues different approaches to that problem” (Kitcher 2011, p. 193). Those who participate in debates on the ethics of reproductive technologies and who employ the arguments outlined above are often philosophers, theologians, sociologists, biotechnologists and so forth; they are rarely historians.

Before delving into the work of historians of eugenics and their influence on debates on reproductive technologies and practices, it is necessary to give a short statement on the structure of the paper. In the next section, I present the work of historians of eugenics and discuss how they have tried to bring to light the multiplicity of practices, policies and actors that characterised twentieth century eugenics. Next, I focus on the meanings of the word ‘eugenics’ and present some of the definitions which are used in debates on reproductive technologies to describe this phenomenon. I identify different strategies to describe eugenics and criticise the use of definitions that presuppose its moral wrongness. I then turn to what I define as the discontinuity and continuity views of the relationship between past and present. I discuss both views and show that they rely on different assessments of what was wrong in the past and that these assessments of the past play an important role in authors’ assessments of the present. My hope is that reflecting on the meanings of ‘eugenics’, on the relationship between past and present, and on the roles and the understandings of eugenics will shed some light on its shadow and contribute to debates on the ethics of reproductive technologies and practices.

### Where are historians when we need them?

In the comparison of reproductive technologies and practices to a historical phenomenon, eugenics, historians could help settle at least *some* of the questions that cause the moral disagreement among scholars participating in debates on their ethical standing, such as whether the similarities between past and present are so significant that the comparison is warranted. Many historians have indeed tried to make sense of the history of eugenics and to reconstruct it while taking into account its complexities, divergences and multifaceted aspects. It is therefore surprising that in debates on the ethics of reproductive technologies, the comparison with this past phenomenon is often made without reference to studies of the history of eugenics, and that the homogeneity of this past phenomenon is often taken as a given. For instance, Bennett (2014) calls Harris’ and Savulescu’s arguments in favour of using PGD to create the best possible child a “eugenic vision”. Despite this, she fails to specify what she means by ‘eugenic’, to refer to the historical unfolding of this phenomenon and to its relationship with PGD and with the work of both Harris and Savulescu. Similarly, Savulescu and Kahane (2009), in their seminal work on procreative ethics and PGD, refer to eugenics in terms of “moral atrocities” and of “the collectivist, coercive and often racist projects of the twentieth century” and conclude that the procreative principles that they have discussed “bear little resemblance” with eugenics. Again, eugenics as a historical phenomenon is a point of reference devoid of its historical unfolding. As Koch (2004) argues:

[T]he witless reference to ‘eugenics’ with no further specification is empty and more often a function of our own projections and intentions than a reference to history. (Koch 2004, p. 329)

Historians and science and technology studies scholars have shown how eugenics, throughout history, cannot be easily reduced to a uniform set of practices and to a univocal ideology (Bashford 2010; Bashford and Levine 2010; Ekberg 2007; Kevles 1985; Koch 2006a; Meloni 2016; Lombardo 2008, 2011; Paul 1984, 1992). Eugenics encompassed a diverse set of practices that included not only race-based segregations and the institutionalisation and (at worst) the killing of the ‘feeble-minded’, but also the development of public health and sexual hygiene programmes aimed at improving environmental conditions (Gyngell and Selgelid 2016), education programmes aimed at spreading eugenic ideas; contests for the ‘fittest’ American family and campaigns for women’s right to abortion and access to contraception (Roberts 1997). It encompassed a wide range of policies such as the Immigration Restriction Act in the US, sterilisation laws in the US, Scandinavian and other countries, but also the legalisation of abortion in some of these countries (with the exception of for instance Norway) (Koch 2006a). It involved a variety of actors belonging to different political parties and embracing different ideologies (Kevles 1985; Meloni 2016; Paul 1984; Roberts 1997), from conservative defenders of the status quo to feminists campaigning for reproductive rights and from socialists and liberal democrats to racist right wingers. It was grounded in “epistemically pluralistic” theories of heredity, with both Lamarckian and Mendelian views influencing eugenic thinking (Gyngell and Selgelid 2016; Meloni 2016, p. 74; Schneider 1990), and both “soft” and “hard” theories of heredity justifying its principles (Meloni 2016, p. 65). It also involved the creation of different institutions such as the British Eugenics Society, the US Eugenics Record Office and the Mexican Eugenics Society. Contemporary historians (Adams 1990; Bashford 2010; Bashford and Levine 2010; Ekberg 2007; Kevles 1985; Koch 2006a; Lombardo 2008, 2011; Paul 1984, 1992) have set out not only to trace this history but also to show that eugenics was not confined geographically to Germany and North America and historically to the years immediately before, during and after World War II, but rather to a much more encompassing period of time and to diverse geographical areas: including Latin America, Central, Eastern and Northern Europe, and China. Differing views of the science of heredity (Gyngell and Selgelid 2016; Meloni 2016) and socio-political contexts (Roberts 1997) gave rise to a differing set of concerns, interventions and policies among geographical regions. For instance, as Roberts (1997) shows, in North America eugenicists and feminists (such as Margaret Sanger, who advocated for birth control measures) formed allegiances as the former “gave the birth control movement a national mission and the authority of a reputable science” (Roberts 1997, p. 72), thereby inspiring policies in line with North America’s focus on controlling reproduction. Lamarckian and soft theories of heredity inspired eugenics programmes in Latin America and the ideas of British thinkers from the left (Gyngell and Selgelid 2016; Paul 1984, 2006), giving rise (in Latin America) to programmes aimed at “improving environmental conditions that influence transmissible (acquired) traits” (Gyngell and Selgelid 2016, p. 148).

Despite the differences among policies, actors, countries and periods of time, some of the features of twentieth century eugenics common across time, space and political affiliations tend to emerge in contemporary discourses on these technologies and practices. These shared features of eugenics^4^ are best identified in eugenics as an ideology (i.e. a set of ideas and beliefs) rather than in eugenics as a practice (i.e. laws, institutions and eugenic education). Eugenics as a practice was a rather heterogeneous phenomenon, but it is possible to identify a core: one that it is shared over time, space and political affiliation.^5^ This core was a concern with improving the quality of the population by preserving some human features considered beneficial for the collective and to avoid, or at least reduce, the transmission of negative features.^6^ The etymological definition of eugenics and the definition formulated by Sir Francis Galton, the “father” of eugenics, capture these shared features. Etymologically, eugenics is composed of the Greek prefix ‘eu’ that translates as ‘good’ and the Greek word ‘genos’ that means ‘birth’ or ‘ancestry’. Galton’s original definition dates back to 1883, when he defined eugenics as:

*The science of improving stock*—not only by judicious mating, but whatever tends to give the more suitable races or strains of blood a better chance of prevailing over the less suitable than they otherwise would have had. ([emphasis added] Galton 1883)

These few features of eugenics as an ideology taken together represent the core of eugenics or, following Meloni (2016), the “common ethos” of eugenics, but it is still difficult to draw lessons on which to base ethical assessments of reproductive technologies and practices (Bashford 2010; Paul 1998). When critics and proponents of reproductive technologies and practices warn that we should be wary of “going back” to eugenics or that we should be very careful not to duplicate it, it is not always clear what they mean; as Paul (1998) puts it: “we’re warned against nothing in particular” (Paul 1998, p. 98).

Due to this complexity, and the emotional power that eugenics has, Wilkinson (2008), Camporesi (2014) and others working in the field of bioethics have suggested that reference to it ought to be abandoned, or at least significantly limited, in debates on reproductive technologies and practices. Their pleas, as well as contemporary efforts to distinguish between ‘good’ and ‘bad’ forms of eugenics, have not really changed current discourses on assisted reproduction as “the identification of a policy or practice as eugenic remains highly stigmatizing” (Paul 1998, p. 261) and references to eugenics in this context continue to abound.

So, historians are there and we need them. They have significantly helped to shed light on the history of eugenics and on the understanding of this phenomenon, on its complexity and multifaceted character. Depending on which aspects of this multifaceted history contemporary authors, policy-makers, journalists, activists and other members of the public look at, different lessons can be drawn and different strategies can be used to discuss, report, regulate, reject or defend reproductive technologies and practices. If eugenics is only depicted in terms of coercion or a quest for perfection (and assuming that both these practices are really ethically troubling), then what today’s technologies and practices need to avoid is to be driven by the latter and organised in terms of the former. If, instead, eugenics was really characterised by differing views of heredity, ideologies, objectives, policies and practices then its lessons are much less straightforwardly derived (Buchanan et al. 2001). Despite this, I would regard knowing a comprehensive account of the history of eugenics as an ethical practice.^7^ It is an ethical practice as on the one hand such knowledge can foster approaches, debates, interventions and policies that are not only mindful of what went wrong in the past, but also try to prevent (and perhaps redress) similar unfoldings. On the other, it can foster debates that are critical of and reflexive towards the social and political contexts in which they take shape and considerations of how both good and bad intentions can lead to undesirable states of affairs.^8^

What all the historical analyses have not managed to do is put to rest contemporary disagreement on what present technologies and practices count as eugenics and whether the similarity between past and present is a sufficient condition to settle the question of their ethical standing and value (Wilkinson and Garrard 2013). What *role* does the shadow of eugenics play within debates on reproductive technologies and practices? The reference to eugenics cuts across competing assessments of reproductive technologies and is used in different ways to create ethical boundaries between acceptable and unacceptable technologies, and their acceptable and unacceptable uses. Within these debates, recurrent questions are whether these technologies and practices amount to eugenics and/or whether they will lead us back to eugenics. However, if we aspire to move towards fruitful debates on the ethics of these technologies, I argue that our attention should be directed elsewhere. An alternative strategy to use in debates on the value and ethical standing of reproductive technologies is to look into the shadow of eugenics and uncover the relationship between past and present, how diverging interpretations of past practices inform our understanding of the present, and how they influence the contemporary disagreements concerning the ethics of reproductive technologies and practices. Looking into the shadow could, in other words, help those who participate in these debates to ask the right questions in order to collectively make progress both in the ethical assessment of these technologies and practices as well as in ethical debates on these technologies more generally.

Foucault, and Socrates before him, taught us that it is worth bearing in mind the importance of taxonomy and of reflecting on meanings as powerful analytical tools to interpret the complexities of reality. Looking into the meanings of eugenics is of interest as a conceptual tool to interpret the present. As Paul (1992) argued:

Eugenics is a word with nasty connotations but an indeterminate meaning. Indeed, *it often reveals more about its user’s attitudes* than the policies, practices, intentions, or consequences labelled. ([emphasis added] Paul 1992, p. 665)

In the remainder of this paper, I will explore two questions: the role of the meanings and the uses of the word ‘eugenics’ in debates on reproductive technologies and practices, and the role of assessments of the history of eugenics (and the lack thereof) in these debates. It is informed by an extensive review of publications that refer to eugenics to strengthen and ground arguments on the ethics of such technologies and practices.

## What’s in the name ‘eugenics’?

In contemporary debates on the ethics of reproductive technologies and practices, the word ‘eugenics’ is defined in a multiplicity of ways. The disagreement regarding the meaning of ‘eugenics’ is not limited to what definition is the most appropriate and why, but rather it centres on “what counts as eugenics” (Wilkinson and Garrard 2013, p. 2), i.e. on which reproductive technologies and practices can be classified as eugenics, and whether this classification can settle the ethical questions that they raise. This section of the paper focuses on the role of the meanings, descriptions and definitions ascribed to eugenics in contemporary debates on reproductive technologies and practices.

One strategy to define the word ‘eugenics’ or to describe this phenomenon^9^ within these debates would be to rely on a definition that is as descriptively accurate as possible, i.e. one that goes beyond the multiplicity of practices, ideologies and actors to capture the shared features of this multiplicity and that strives for neutrality in that it tries not to presuppose any explicit negative or implicit built-in value-judgment.^10^ An example of such a definition of ‘eugenics’ would be: “the attempt to influence the genetic endowment of future generations”.^11^ Many of the authors who refer to eugenics in debates on the ethics of reproductive technologies adopt this strategy and rely on this type of definition of eugenics.^12^ For example, Anomaly (2014) describes eugenics as: “any attempt to harness the power of reproduction to influence the genetic composition of future people” (Anomaly 2014, p. 179). Similarly, Glover (2006) argues that eugenics can be understood “broadly”^13^ as “any decisions, including parental decisions, about what sort of child will be born” (Glover 2006, p. 28). As I discuss in the next section, most of the authors that adopt this strategy (and most authors in general) do condemn eugenics, but they are also broadly in favour of reproductive technologies.^14^

A second strategy adopted by those who refer to eugenics in debates on reproductive technologies and practices is to rely on a definition or description of it that incorporates background ethical assumptions on the (negative) ethical standing of eugenics. For instance, Garland-Thompson (2012) describes “eugenic logic” as aiming to “eliminate disability and, by extension, disabled people from the world” (Garland-Thompson 2012, p. 340). Authors who oppose the use of CRISPR for germline editing and of MRTs associate eugenics with these practices (Brokowski et al. 2015; Darnovsky 2013). Similarly, in authors who condemn human enhancement, eugenics becomes synonymous with enhancement (and equally condemned) and the antonym of treatment (Habermas 2003; Sandel 2004). Whether disability should be eliminated, or whether germline editing and human enhancement should be pursued, is a matter of contention in bioethics, as debates on the ethics of human enhancement,^15^ on the ethics of screening technologies that allow to select against disability^16^ and on the ethics of germline editing^17^ show. My claim here is that these descriptions and definitions incorporate background ethical assumptions against germline editing, enhancement or against building a disability-free world and on the—related—ethical standing of eugenics. Hence, they opt for a different strategy to define and describe eugenics from the first group of authors described above.^18^

Therefore, even if authors tend to agree on the negative connotations of ‘eugenics’, they opt for different strategies to define this word. But which strategy should be preferred? Should we opt for descriptive accuracy or for a definition that conveys a message that expresses one’s own moral beliefs on the wrongness of eugenics? Or, again, should the word ‘eugenics’ be employed at all? As often, the answer to these questions depends on what one wants to achieve by using this word.

### Descriptive accuracy or conveying a message?

Many who refer to eugenics in debates on reproductive technologies either use a comparison with the past to show that such technologies are similar to eugenics and hence as morally problematic (the argument underlying the continuity view) or to show that these technologies are different from eugenics and hence not as morally problematic as eugenics was (the argument underlying the discontinuity view). In both types of arguments, the reference to eugenics is used to support one’s position on the ethics of the reproductive technology or practice in question. It has, in other words, a normative role. Considering that both proponents and critics of reproductive technologies and practices agree on the negative connotations of eugenics, it may seem *prima facie* that it does not matter which definition they employ. They can say that eugenics is an attempt to improve the human gene pool or that it is an attempt to eliminate disabled people, and it would not matter for their arguments on contemporary technologies because that is where the moral disagreement lies. But words, metaphors and rhetoric matter greatly in these debates (O’Keefe et al. 2015; Ravitsky et al. 2015). As Blackburn (1998) argues: “words typically nudge people, with more or less subtlety, towards attitudes to the things they pick out” (Blackburn 1998, p. 15), and they can redirect people’s interests (Stevenson 1937). Employing the word ‘eugenics’ and a certain definition of it has normative implications: as Wilkinson (2008) shows and as others argue (Camporesi 2014; Gillon 1998; Paul 1998), the *use* of the word ‘eugenics’ in contemporary debates on reproductive technologies has significant implications due to its emotive power and negative connotations.

Studies in moral psychology have provided evidence for how wording, context and order have *framing effects,*^19^ namely they influence people’s moral judgements on different matters (Haidt and Baron 1996; Haidt and Björklund 2007; Lakoff 2004; Petrinovich and O’Neill 1996; Sinnott-Armstrong 2007). A person’s (moral) beliefs would be subjected to a word-type framing effect when “whether [or not] the person holds the belief depends on *which words are used to describe* what the belief is about” ([emphasis added] Sinnott-Armstrong 2007, p. 52) rather than on what the belief is actually about. So, a person’s intuitions are subjected to framing effects if their moral beliefs regarding a given reproductive technology depend on the way the technology is described, on *which kind of words* are used to describe it rather than on the technology and its applications. Choosing one type of definition over another matters normatively because it can influence people’s moral judgements concerning reproductive technologies and practices, and hence the decision to employ one type of definition or another is not per se neutral (Lakoff 2004).

Wilkinson (2008) argues that the word ‘eugenics’ should not be used in debates on the ethics of selective reproductive technologies due to its emotive power and negative connotations. According to this author, the word ‘eugenics’ has the potential to unleash negative emotions that can “circumvent or neutralise people’s critical-rational faculties” (Wilkinson 2008, p. 470) and cloud their judgement about the reproductive technology or practice being discussed.^20^ Hence the word ‘eugenics’ should not be brought up because it fails to respect the autonomy of those who engage with these debates^21^ (Wilkinson 2008), because it is descriptively inaccurate and because it does not add anything in terms of conceptual clarity (Camporesi 2014; Gillon 1998). I am sympathetic to such analyses and certainly in favour of conceptual clarity and of avoiding misleading and factually wrong^22^ references to historical events. ‘Eugenics’ is indeed used as the “*reductio ad Hitlerum*” described by Strauss (1953) whereby a person or a practice becomes guilty by virtue of their association with the Nazis (Strauss 1953). The comparison between reproductive technologies and historical eugenics is often used to condemn by association these technologies.

Despite this, it seems odd that the best strategy to protect people’s rational capacities is to deliberately avoid the use of a word, even a heavily emotively-loaded word. There are different reasons why the word eugenics features in association with reproductive technologies: it may be that the user believes that these technologies are similar to eugenics or at least that they are comparable to it in meaningful ways; or it may be that the user is motivated by eugenics’ persuasive power and its potential to elicit negative assessments of reproductive technologies. In the former case, what matters is whether the user is factually wrong or not; in the latter, it matters what rules of moral argumentation are set in debates on reproductive technologies and practices.

As the stakes are high (we are talking about the ethical assessment of reproductive technologies and practices), it is reasonable to state that one should be careful about how to use the word ‘eugenics’ and how one chooses to define it. Hence, I would suggest that, contrary to what Wilkinson (2008) and others argue, we should aim to adopt a reflective approach to the use of the word eugenics rather than to make it taboo. We should aim for conceptual clarity, for definitions that are as descriptively accurate as possible, that fairly represent what eugenics encompassed, and that are informed by the work of historians of eugenics. A description-oriented definition of ‘eugenics’ would allow us to start with a common ground to discuss both the history of eugenics and the ethics of reproductive technologies.

There are different reasons why those who participate in debates on the ethics of reproductive technologies should, where possible, avoid definitions of ‘eugenics’ which are fraught with negative connotations and that conflate descriptive and evaluative elements. These definitions serve the normative goal of critics of reproductive technologies (i.e. elicit negative judgements of these technologies), but do not improve the ethical debate insofar as they turn the attention to whether these technologies are eugenics rather than on relevant moral aspects of these technologies. They pre-determine the moral questions and hence shape the debate by deciding beforehand which aspects should be given attention and which are irrelevant (Jasanoff et al. 2015). They pre-determine what ethical questions should be discussed and what direction the ethical debate should take, thereby excluding views that do not fit within the pre-established framework. They add ethical complexity to already complex questions, and they do not provide those participating in the debate with a way to make sense of this complexity. Regardless of one’s normative goal, then, intellectual honesty would call for definitions, meanings and uses of the word ‘eugenics’ which are as descriptively accurate as possible. Only in this way can we really assess whether the comparison with past and present is warranted and make steps forward in the ethical debate on reproductive technologies.

## The discontinuity and continuity views

Let me take a moment to recall the form taken by two commonly used arguments within debates on the ethics of reproductive technologies and practices which involve references to eugenics. The first set of arguments that stress the discontinuity between past and present goes something like this:

1. x, y and z are morally wrong acts;
2. eugenic practices entailed x, y and z;
3. old eugenic practices were morally wrong.
4. a (a reproductive technology or practice) does not entail carrying out x, y and z;
5. other things being equal, a is not morally wrong.

The opposing set of arguments, which stress elements of continuity between past and present, runs something like this:

1. x, y and z are morally wrong acts;
2. eugenic practices entail x, y and z;
3. eugenic practices are morally wrong.
4. a (a reproductive technology or practice) entails x, y and z;
5. a is morally wrong.

Throughout the rest of the paper, I refer to these two views as the discontinuity view and the continuity view of past and present. My argument is that the understanding of the history of eugenics and the features of the history upon which one focuses are deeply interlinked with the claims that one makes about the ethics of reproductive technologies.

### The discontinuity view

Those who hold the discontinuity view condemn characteristics of eugenics concerning its scientific foundations. More specifically, they hold that eugenics was informed by a limited knowledge of the science of heredity (Epstein 2003; Glover 2006), that it did not meet appropriate ethical and scientific standards of research (Appel 2012; Tong 2013), and that it mistakenly relied on the belief that social, behavioural and ethnic features could be flattened and reduced to mere biological dimensions (Buchanan et al. 2001; Scott 2006). A second problematic feature of eugenics is identified in its underlying racist and discriminatory beliefs, and in the policies that these beliefs inspired (Agar 2008; Buchanan et al. 2001; Robertson 2005; Savulescu and Kahane 2009; Scott 2006). The authors defending this view largely focus their attention on North American immigration policies designed to restrict incomers from certain ethnic groups (i.e. Southern and Eastern Europe), on American sterilisation policies that targeted people with physical and mental disabilities and members of lower socio-economical classes (Appel 2012; Crossley and Shepherd 2003), and on Nazi eugenics aimed at creating a “master race” through the elimination of people with disabilities (Blackford 2005; Glover 2006; Walker 2010).

In addition to bad science and discriminatory beliefs, in debates on reproductive technologies and practices, coercion becomes the capital sin of past eugenics^23^ (Agar 2008; Bruni et al. 2012; Caplan et al. 1999; Crossley and Shepherd 2003; Glover 2006; Robertson 2005; Santosuosso et al. 2007; Savulescu and Kahane 2009). While agreeing that coercion was one of the most problematic elements of eugenics, authors discussing this feature focus on slightly different features of it: some criticise state interference in the realm of reproduction and the denial of what today is defined as “procreative liberty”^24^ (Robertson 2005). Others mainly address the question of exercising control over biological features of the population (Bouffard et al. 2009; Dolgin 2004), whereas another group sees in coercion a denial of the respect for individuals’ bodily integrity (Appel 2012; Santosuosso et al. 2007). A final reason why eugenics is perceived as despicable is that its policies and aims were oriented towards the improvement of the wellbeing of the population rather than the good of the individuals (Fenton 2006; Glover 2006; Robertson 2005; Savulescu 2005; Scott 2006). In all these references to the past, despite some internal differences concerning the most contemptible elements of eugenics, old eugenics is unanimously condemned. What varies is the weight that should be assigned to ‘x, y, z’, i.e. to each of the features of eugenics on which scholars participating in debates on the ethics of reproductive technologies focus. The discontinuity view underlines the idea that if reproductive technologies and practices do not entail ‘x, y, z’, namely coercion, bad science, discriminatory beliefs and a precedence of population-concerns over individual-concerns, *then* they are not ethically troubling in the same way as eugenics.

Authors relying on the discontinuity view to support their disanalogy between the past and present argument make slightly different claims about the present and about the relationship between past and present. For instance, some argue that past eugenics and reproductive technologies do not share any significant feature (Bourne et al. 2012; Savulescu and Kahane 2009). These authors argue that reproductive technologies are guided by values and inspired by moral beliefs that have nothing to do with those of eugenics: while eugenics was discriminatory and exclusionist, reproductive technologies are value-neutral with respect to race, gender and class, and oriented towards the welfare of the future child or designed to enhance autonomous decision-making (Glover 2006; Harris 2007; Savulescu and Kahane 2009; Robertson 2005). In their accounts, past and present differ in terms of both the underpinning values and the means employed: while eugenics was coercive, violent, and entailed forced sterilisations and mass killings, today’s reproductive technologies are freely chosen, do not entail gruesome methods and are available to those who wish to use them (Appel 2012; Bourne et al. 2012; Robertson 2005; Savulescu and Kahane 2009).

Other authors allow that although these technologies share features with past eugenics, they are still free of the characteristics that made eugenics morally wrong (Agar 2008; Camporesi 2014; Fenton 2006; Harris 1993; Scott 2006; Wilkinson 2010). For instance, Wilkinson (2010) reflects on the question of whether “the very idea of ‘genetic improvement’ is a mistake” (Wilkinson 2010, p. 159) and concludes that the answer to that question, once we add some qualifications (which he offers in his book), is negative:

There have been many versions of ‘eugenics’ that have incorporated dangerously flawed ideological and pseudo-scientific beliefs, *such as Nazi racial ‘science’*. However, there is no need to assume that all attempts to improve the ‘gene pool’ will be similarly flawed […]. So perhaps (for example) improving the gene pool in ways that improve future public health would be morally acceptable (and even desirable) ([emphasis added] Wilkinson 2010, p. 166)

Similarly, Harris (1993), in addressing the question of whether gene therapy should be considered a form of eugenics, argues that if one relies on an understanding of eugenics akin to the understanding that its father, Francis Galton, had, then the answer is positive. Eugenics and gene therapy have a common aim: they both seek to produce “fine children” (Harris 1993), an aim that, in the eyes of the author, is considered worthwhile. This aim is worth pursuing both in the case of attempting to “remove or repair dysfunction” and in the case of “measures designed to enhance function”^25^ (Harris 1993). These authors (and other sharing their view such as Agar 2008) rely on a broader understanding of eugenics as the attempt to improve the gene pool of the population and argue that what was problematic in the past was *how* eugenicists tried to achieve human improvement, i.e. the relying on coercive and violent measures, rather than on the aim of eugenics and its underpinning values per se.

### The continuity view

The authors who defend the continuity view broadly agree with those defending the discontinuity view with respects to the condemnable features of eugenics. For instance, some scholars criticise its faulty scientific foundations and the quest for singling out biological components of social characteristics (Jeffreys 2012); others focus on the coercive character of eugenics’ policies and practices (Epstein 2003), their incorporation into the political agenda (Hampton 2005; Roberts 2009), their violation of bodily integrity and of reproductive freedom (Epstein 2003; Roberts 1997). Despite the similarities between those defending the discontinuity and the continuity view in terms of the condemned features of eugenics, the degree to which these features are considered problematic and the moral weight given to each feature differ substantially. Contrary to those who defend the discontinuity view, most of the authors defending the continuity view do not see in coercion and state-driven practices the capital sins of eugenics. They instead focus on eugenics’ discriminatory attitudes, on its morally wrong underpinning values, on the policies that were designed following these values, and on the effects on minorities and vulnerable groups of these attitudes, values and policies (Garland-Thomson 2012; Hampton 2005; MacKellar and Bechtel 2014; Roberts 1997, 2009; Rosen 2003; Sparrow 2011a). They also stress eugenics’ ‘unhealthy’ preoccupation with perfection (Bashford 2010) and argue that eugenic practices of the past were ultimately driven by the purpose of perfecting the population. This aim is considered problematic not because—as the defenders of the discontinuity view would argue—their efforts were directed at the population rather than at the individual, but because of the immorality of the aim itself (MacKellar and Bechtel 2014; Rosen 2003; Sandel 2004).

Commentators agree on the wrongness of most practices within twentieth century eugenics, on the aberrant means used to pursue its aims and on the need to avoid repeating these mistakes, but they draw different lessons from these analyses, and they develop competing assessments of contemporary reproductive technologies and practices. The reason for this, I argue, is that their views diverge on the underpinning values of eugenics (such as the desirability of influencing the genetic endowment of future generations) and to the weight that is given to each condemned feature of twentieth century eugenics. The moral disagreement on the present has roots in a disagreement about the past,^26^ and past and present are profoundly interlinked in these accounts.

Authors supporting the continuity view present a number of reasons to draw attention to the meaningful similarities between contemporary technologies and practices, and features of twentieth century eugenics. For instance, some stress that the (cumulative) effects of these practices match some of the effects sought by eugenicists or some of the effects eugenic policies sought to engender. Screening technologies such as PGD and PND, but also new reproductive technologies such as MRTs, will contribute to a decrease in the number of disabled people and to members of ethnic minorities (Hampton 2005; Garland-Thomson 2012; de Melo-Martín 2016; Roberts 2009). This decrease is considered by these commentators to be not only ethically troubling in itself, but also for consequentialist reasons, i.e. for the effects that it will have on these groups. The fear is that members of certain ethic groups (Roberts 1997, 2015; Russell 2010), women (de Melo-Martín 2016; Epstein 2003) and disabled people (Garland-Thomson 2012) will be increasingly stigmatised, as they were in the past, and publicly funded services available to them will be reduced (Garland-Thomson 2012; Scully 2008; Sparrow 2008, 2011b).

While authors defending the continuity view do not see in coercion one of the chief wrongs of eugenics, they still condemn it and argue that some elements of coercion survive in contemporary reproductive technologies and practices. Unlike supporters of the discontinuity view, these authors stress that the alleged diminished intervention of the state in matters of procreation is either a misrepresentation of the present situation or a sign that coercion is understood in an unduly narrow sense. For instance, Sparrow (2008) argues that the fact that certain screening technologies are “made available” signals the continuity of state interventions in matters of procreation. Not only that, but according to other critics of reproductive technologies and practices that refer to eugenics, coercion is an element of today’s technologies and practices even if the state does not have an active role in promoting them (Mehlman 2011; Mittra 2007; Sparrow 2011b). Following these authors, other than from direct state interventions, coercion may result from the pressure to use reproductive and screening technologies exercised by healthcare professionals (Ekberg 2007; Koch 2006b), scientists (Darnovsky 2004; Ekberg 2007) and bioethicists (Koch 2006b). Other than coercion, what troubles defenders of the continuity view about eugenics is the quest for perfection that it entailed. In their view, this quest is embodied by reproductive technologies and practices (Sandel 2004).

## Learning and moving forward

I started this paper by arguing that historians have reconstructed the unfolding of eugenics and brought to light the multiplicity of themes, policies, actors and values which it encompassed. Learning from history in order not to repeat the mistakes of the past is a noble, and some would argue a necessary, endeavour. But to learn from history, history must be known. Partial or inexistent historical accounts make it extremely difficult to learn from the past and, as Bashford (2010) puts it:

Selective understandings of the history of eugenics may seriously mislead contemporary efforts to regulate reproductive and genetic technologies, and be a questionable basis for policy decisions. (Bashford 2010)

As I showed in the previous sections, despite unanimously condemning eugenics, defenders of the discontinuity and continuity views focus their attention on slightly different ethically troubling features of the past. While defenders of the discontinuity view see coercion and population-wide eugenic policies and practices as the most problematic feature of twentieth century eugenics, defenders of the continuity view see the callous attitudes towards disabled people and other minorities, and the drive towards improving the gene pool of the population, as the most despicable feature of eugenics. These differing ethical assessments of the past are linked with differing ethical assessments of the present: defenders of the discontinuity view stress how reproductive technologies and practices both promote and protect reproductive freedom and individual welfare (as opposed to population-wide approaches) and individual autonomy (as opposed to coercion). They see in the promotion and protection of these values the most salient characteristics of reproductive technologies and practices, and the reasons why they should be welcomed. On the contrary, defenders of the continuity view stress how reproductive technologies and practices both embody and play a role in the persistence of the drive towards perfecting the population and of the discriminatory attitudes towards women, disabled and black people, and the reasons why they should be condemned. They see in this condemnation and problematisation of these technologies and practices a means to promote different values. These differing ethical assessments of the past can also explain why proponents of the discontinuity view often dismiss concerns about the present expressed by those who support the continuity view. They dismiss them as signals of conservative attitudes towards new reproductive technologies and practices and of a poor understanding of today’s effects and uses of these technologies and practices. However, in reality, the two groups condemn and justify different features of the past and of the present.

Historians and critical theorists have warned of the risk of focusing excessively on the coercive character of eugenics whilst ignoring the patterns of coercion and discrimination present in reproductive technologies and practices (Bashford 2010; Ekberg 2007; Roberts 1997). On the one hand, eugenicists advocated voluntary forms of eugenics; on the other, social pressure, discriminatory attitudes and barriers to fully exercise and to have respected one’s reproductive freedom may be (in different ways) features of today’s reproductive technologies too (Bashford 2010; Koch 2006a; Paul 1992). The discontinuity view’s underlying arguments draw a line between historical eugenics as coercive and reproductive technologies as freely chosen by autonomous individuals, but the reality is much more blurred than advocates of such technologies make it out to be. At the same time, authors focusing on the callous attitudes towards disabled people, women and ethnic minorities, and on persisting biases and forms of discrimination enacted within and by reproductive technologies and practices may risk overlooking significant differences from the past in terms of the political and social context in which reproductive technologies and practices are developed. While it is undoubtedly true that despicable attitudes still exist and that they should be resisted, efforts and steps towards fostering respect for these groups, to guarantee them public assistance and to develop means for greater integration, are being made. Additionally, empirical data have shown that parents who make use of reproductive technologies are not driven by a quest towards perfection or by discriminatory beliefs (Franklin and Roberts 2006; Kerr 2004).

Once history—in all its complexities, nuances, peculiarities—is known, we can learn from it. We can start with a common ground that avoids misleading assessments and misleading conclusions. Despite this, such knowledge about history cannot solve the moral disagreement concerning what underpinning values are worth pursuing and what others are worth dismissing. It cannot answer, in other words, the question of which aims and values reproductive technologies and practices should serve. History can tell us that coercion was not the only nor the most distinctive feature of eugenics, but it cannot tell us whether trying to improve the gene pool of the population and trying to increase the number of babies born without disabilities are worthy aims (as most defenders of reproductive technologies argue). Similarly, it can tell us about eugenic policies and how those policies contributed to an increased stigmatisation of disabled people and of the perceived need to wipe them out, but what it cannot tell us is whether allowing gene editing technologies will lead prospective parents to select blond blue-eyed babies and whether this is something that should be opposed.

For these reasons, while it is important to learn *about* the history of eugenics and learn *from* the history of eugenics, this is probably all that eugenics should contribute to contemporary debates on reproductive technologies. Mainly focusing on eugenics and on analogies/disanalogies between past and present risk jeopardising contemporary debates on the ethics of reproductive technologies and shifting the focus away from relevant questions about the value of reproductive technologies and practices.

## Conclusions: looking into the shadow

In this paper, I have discussed how the word ‘eugenics’ and the history of eugenics are used in debates on the ethics of reproductive technologies and practices. I have showed that some commentators employ definitions of ‘eugenics’ which are descriptively accurate while others opt for definitions which immediately bring to the fore the negative connotations of this phenomenon. I have argued that, when possible, the former type of definition should be preferred over the latter. I then moved on from a discussion of the uses of the word to the uses of the history of eugenics and showed how authors who focus on certain problematic characteristics of the past tend to have views on contemporary reproductive technologies and practices that are symmetric with, and a response to, these characteristics.

The shadow of eugenics extends to contemporary reproductive technologies and practices and it is a legacy that will probably be hard to eradicate, and it might not even be desirable to do so. Scholars who participate in debates on these technologies should learn about the historical unfolding of eugenics in order to avoid repeating the same mistakes that were committed in the past.

## Footnotes

1 Throughout the paper, if I am not referring to a specific technology or practice, I refer to all of them collectively as ‘reproductive technologies or practices’.

2 In addition to being informed about these technologies’ and practices’ technical characteristics, their potential applications, safety, efficacy, etc.

3 With notable exceptions. See for instance: Buchanan (2007), Buchanan et al. (2001), Camporesi (2014), Gyngell and Selgelid (2016), Selgelid (2000), Wikler (1999).

4 For an interesting analysis of eugenic ideas throughout Western philosophical and political thought, see Häyry (2008).

5 Concerning this, Meloni (2016) argues that “the varieties of eugenics in the first decades of the twentieth century were united less by direct politicization of a particular theory of heredity than by a *common ethos*, which crossed over the linguistic and aesthetic borders dividing eugenic approaches” ([emphasis in original] Meloni 2016, p. 66). The author identifies four main features of this “common ethos”, namely: radical biologism, utopian social engineering, “unlimited empowerment of scientific experts”, and the primacy of the race over the individual (Meloni 2016, pp. 66–67).

6 In this paper, I do not focus on or assess historical features of eugenics as my aim is to reflect on the relationship between past and present, and on how past eugenics is used in contemporary ethical debates. For detailed accounts of the history, see for instance: Adams (1990), Bashford and Levine (2010), Kevles (1985), Lombardo (2008, 2011).

7 For an interesting discussion of the relationship between history and bioethics and of how these scholarships can be mutually beneficial, see Wilson (2013).

8 Relatedly, Buchanan et al. (2001) concur that different accounts of the history have different implications for today’s policy debates and that if today’s genetics is “eugenics under a different name”, then “we must achieve a clear understanding of the morality of both” (Buchanan et al. 2001, p. 40). See also Wikler (1999) and Gyngell and Selgelid (2016) on the lessons of eugenics.

9 Whether the word ‘eugenics’ should be referred to at all is discussed in the following section. I do not explicitly discuss the proposal of Agar (2008) and others who share his view of rehabilitating the term ‘eugenics’ by adding the adjective ‘liberal’. While this use of the term ‘eugenics’ differs from the uses of many other authors, those who speak about “liberal eugenics” too have to decide which strategy among the ones described in this section they would adopt.

10 A detailed discussion of the question of whether definitions can be entirely neutral, i.e. whether they can avoid *any* implicit or explicit value judgement is beyond the scope of this paper. I am personally sceptical with respect to the possibility of complete value-neutrality in definitions and descriptions of technologies and practices that are so fraught with emotive meanings, if only because definitions are formulated by “moral animals” (Wright 1994): human beings with values and moral beliefs. For the present purpose, it is sufficient to say that whilst complete neutrality might be unattainable (Lakoff 2004; Stevenson 1937), the degree to which this can be attempted varies greatly, and one could opt for a definition that is as descriptively accurate as possible.

11 This sentence captures the core of different definitions found in the literature which rely on the strategy described above, but the exact terminology might vary from publication to publication.

12 What I would consider a subset of this group is made by authors who rely on a definition that captures shared features of eugenics (its core) and that is hence descriptively accurate, but which employs the word ‘improvement’ to describe attempts to intervene in and influence the genetic endowment of future generations. For instance, Selgelid (2014) defines ‘eugenics’ “broadly” as: “a practice that aims to improve human lives by employing an understanding of heredity in the exertion of control over who gets born or who reproduces” (Selgelid 2014, p. 3) and Glover (2006) adopts the same strategy (see note below). Defining ‘eugenics’ in terms of improvement entails remaining true to Galton’s original definition, which is arguably an instance of descriptive accuracy. Despite this, defining ‘eugenics’ in terms of *improving* the gene pool (or with its terminological sibling ‘producing “fine children”’, Harris 1993, p. 178) may be interpreted as sneaking in a positive connotation and a built-in value judgement and hence failing the ‘strive for descriptive accuracy and neutrality’ test. I am indebted to César Palacios-González for bringing this to my attention.

13 According to Glover (2006), eugenics can be also understood “narrowly” as: “social policies aimed at improving the gene pool” (Glover 2006, p. 28).

14 Other than the authors mentioned above, examples of authors in favour of new reproductive technologies that define eugenics in descriptively accurate terms are manifold. Just to mention a few of these authors: Agar (2008), Gyngell and Selgelid (2016), Robertson (2005).

15 See for instance: Buchanan (2011), Harris (2007), Sandel (2004), Savulescu and Bostrom (2009).

16 See for instance: Edwards (2004), Parens and Asch (2003), Shakespeare (2006).

17 See for instance, Gyngell et al. (2016), Cavaliere (2018).

18 I am thankful to one of the anonymous reviewers for raising important questions about this section and for pressing me to improve it.

19 Word-framing effects have been studied most notably by Petrinovich and O'Neill (1996) and by Haidt and Björklund (2007). Petrinovich and O'Neill (1996) observed the effects on people’s judgements on a given question being formulated in two different ways (one including the word ‘kill’ and the other including the word ‘save’) to describe two situations which would generate the same consequences. The consequences of the action entailed a certain number of people being killed and a certain number being saved in both situations, but participants were consistently much more likely to act when the word ‘save’ rather than when the word ‘kill’ was employed in the question. They judged the action more morally problematic (and hence they were not keen to act) when the word ‘kill’ appeared in the question. This is a typical example of a word-framing effect, one that works towards redirecting people’s interests as in the case of eugenics and reproductive technologies and practices.

20 Wilkinson (2008) discusses this phenomenon in the context of PGD.

21 According to Wilkinson (2008), the word ‘eugenics’ should be avoided regardless of one’s own moral beliefs on reproductive technologies and practices as “there are some ways of communicating that, without lying or misleading, fail to respect people’s autonomy: methods of communication that circumvent or neutralise people’s critical-rational faculties” (Wilkinson 2008, p. 470). Following this view, which Wilkinson considers “non-partisan” and defines as “the Autonomy argument” against using the word ‘eugenics’, *both* those in favour of and against reproductive technologies have an interest in not referring to the word ‘eugenics’ as both camps should aim for a “rational” debate.

22 With the term ‘factually wrong’, I mean references to the past that do not describe documented events of the past or that deny that certain documented events have taken place.

23 This is also shown by the number of publications which address the question of coercion. In spite of this, most of the authors do not define what they mean by coercion and which features make eugenic policies and practices coercive.

24 Or, alternatively: “reproductive freedom” (Brock 2005; Harris 1992; Cavaliere and Harris 2018).

25 Harris here relies on a critique of the existence of significant moral differences between therapy and enhancement. I will not enter in this debate here, but Harris, among others, does so throughout his work (1992 and 2010).

26 As argued above, the past must be known in all its nuances and complexities in order to assess it from an ethical point of view and for it to be of any interest in present debates.
